# Novel strategy for comprehensive therapy with sustainably complete response in a patient with limited-stage small cell lung cancer: a case report

**DOI:** 10.1177/03000605241305429

**Published:** 2024-12-24

**Authors:** Jianing Jiang, Jinqi Gao, Jing Ben, Gang Wang, Wenqi Duan, Hao Liu, Qianchen Jin, Ruoyu Wang, Jinyan Lv

**Affiliations:** 1 Department of Oncology Medicine, 66562 Affiliated Zhongshan Hospital of Dalian University, Dalian, China; 2 The Key Laboratory of Biomarker High Throughput Screening and Target Translation of Breast and Gastrointestinal Tumor, Liaoning Province, China; 3 Department of Intervention, The Second Hospital Affiliated to Dalian Medical University, Dalian, China

**Keywords:** Limited-stage small cell lung cancer, comprehensive therapy, durvalumab, anlotinib, albumin-bound paclitaxel, complete response

## Abstract

Small cell lung cancer (SCLC) is an aggressive neuroendocrine tumor with the poorest prognosis among all types of lung cancer. Developing an effective comprehensive strategy remains a key focus. We herein present the first documented case of a 68-year-old man with limited-stage SCLC who has maintained a complete response (CR) for over 30 months to date. CR was achieved with first-line chemotherapy using etoposide and carboplatin combined with chest volumetric-modulated arc therapy. Maintenance therapy with anlotinib extended the progression-free survival to 20 months after first-line therapy. When resistance developed, second-line therapy with albumin-bound paclitaxel, carboplatin, and the immune checkpoint inhibitor durvalumab sustained CR for 7 months. Third-line therapy with etoposide and cisplatin combined with durvalumab has maintained CR to date.

## Key message

Using a novel comprehensive therapeutic approach—first-line etoposide and carboplatin combined with volumetric-modulated arc therapy and maintenance antiangiogenic therapy, second-line immunotherapy with albumin-bound paclitaxel and carboplatin, and third-line etoposide and cisplatin with durvalumab—a patient with limited-stage small cell lung cancer has achieved and maintained a complete response for more than 32 months to date.

## Introduction

Small cell lung cancer (SCLC) accounts for 13% to 15% of newly diagnosed lung cancers and has a poorer prognosis than non-small cell lung cancer.^1^ Approximately 40% of patients with SCLC are diagnosed with limited-stage SCLC (LS-SCLC), which has a 5-year survival rate of only 10% to 20%.^2^ Etoposide and cisplatin (EP) combined with radiotherapy has long been the standard first-line treatment. However, this approach achieves cure in only a small fraction of patients, with an overall survival (OS) of 8 to 13 months.^3^ For second-line treatment, the only approved drug is topotecan, which is associated with significant toxicity, a poor response rate of 24%, and a median OS of 6 months.^4^ We herein report the first documented case of a patient with LS-SCLC to achieve long-term survival of 32 months to date.

## Case report

A 68-year-old man visited our hospital with a 1-month history of cough, white frothy sputum, and fever. Physical examination revealed diminished breath sounds and percussive dullness in the left lung, and no other significant findings. The patient had a long history of smoking but no notable medical, family, or psychosocial history. He had not received any prior interventions.

**Figure 1.**
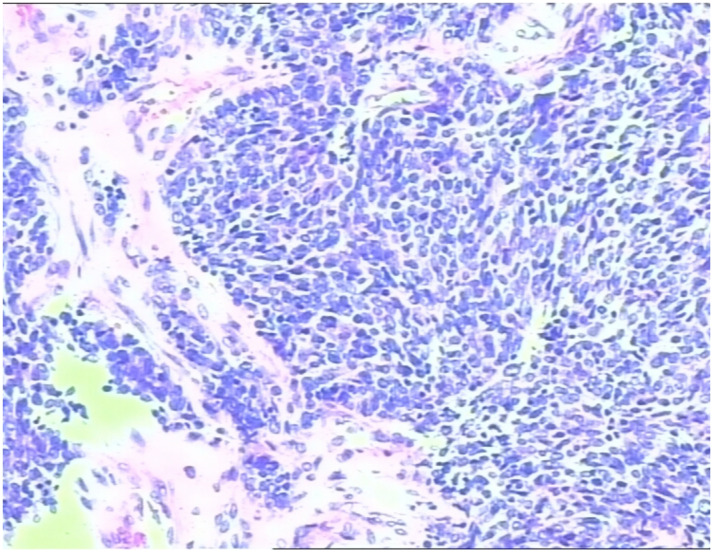
Pathological examination of a peanut-sized foreign body expectorated during hemoptysis revealed neuroendocrine carcinoma cells.

Upon admission, repeat physical examination confirmed reduced respiratory sounds in the left lung and the presence of moist rales. Percussion of the left lung remained dull. Chest computed tomography (CT) showed obstructive inflammation in the lower lobe of the left lung. Fiberoptic bronchoscopy was performed, revealing lymphadenopathy in the left hilar region and left-sided pleural effusion. Initial treatment with antitussive, antiasthmatic, and anti-infective therapy alleviated the symptoms. However, hemoptysis occurred, producing a peanut-sized foreign body, which was identified as a lung neuroendocrine carcinoma by pathological examination (Figure 1).

**Figure 2.**
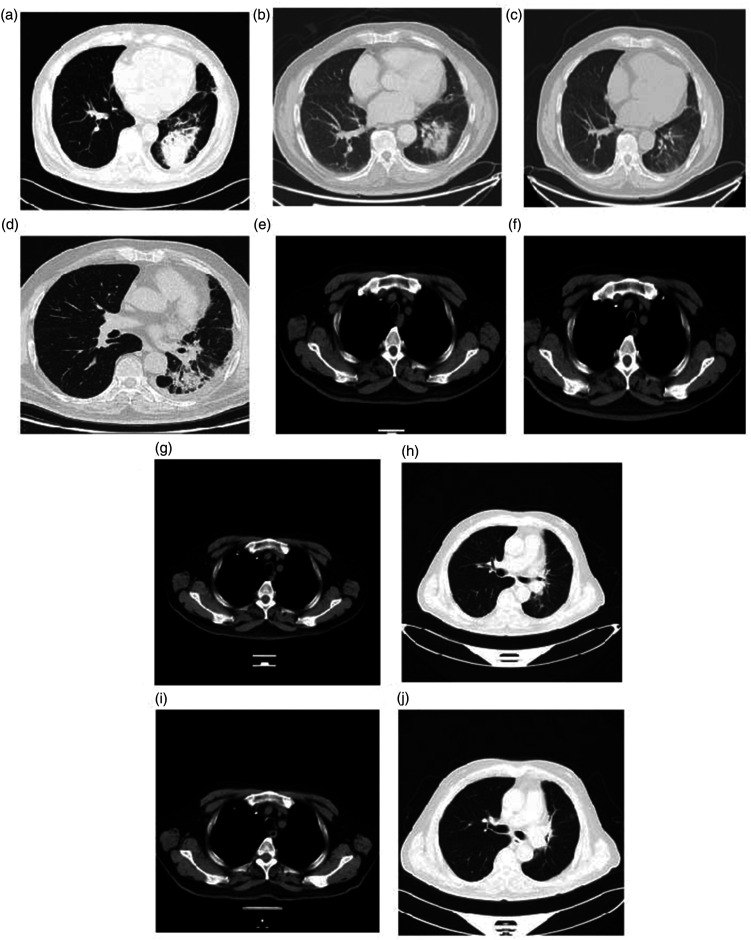
(a) Baseline chest CT scan in August 2019. (b) Chest CT scan after two cycles of etoposide combined with platinum treatment in September 2019. (c) Chest CT scan after four cycles of chemotherapy and CRT in November 2019. (d) Chest CT scan 6 months after CRT showing a new shadow in May 2020. (e) Chest CT showing a new lesion in a mediastinal lymph node in March 2021. (f) Chest CT after two cycles of paclitaxel (albumin-bound) and carboplatin combined with the PD-L1 inhibitor durvalumab in April 2021. Chest CT showing (g) a new lesion in mediastinum and (h) new lesions in the hilum and lower lobe of left lung in October 2021. (i) and (j) Chest CT after four cycles of EP combined with the PD-L1 inhibitor durvalumab in February 2022.

Immunohistochemical analysis demonstrated the following profile: CK (−), vimentin (−), TTF1 (+), CK5/6 (−), CK7 (−), P63 (−), LCA (−), Syn (+), CD56 (+), CgA (+), and a Ki-67 index of 80%. Fiberoptic bronchoscopy further revealed a neoplasm within the basal and dorsal segments of the bronchus, extruding into the lumen and causing stenosis. Laboratory tests showed an elevated neuron-specific enolase (NSE) level of 30.19 ng/mL, whereas the pro-gastrin-releasing-peptide (pro-GRP) level was was normal. Follow-up chest CT showed reduced obstructive inflammation in the lower lobe of the left lung, persistent hilar lymphadenopathy, and left-sided pleural effusion (Figure 2(a)).

**Figure 3.**
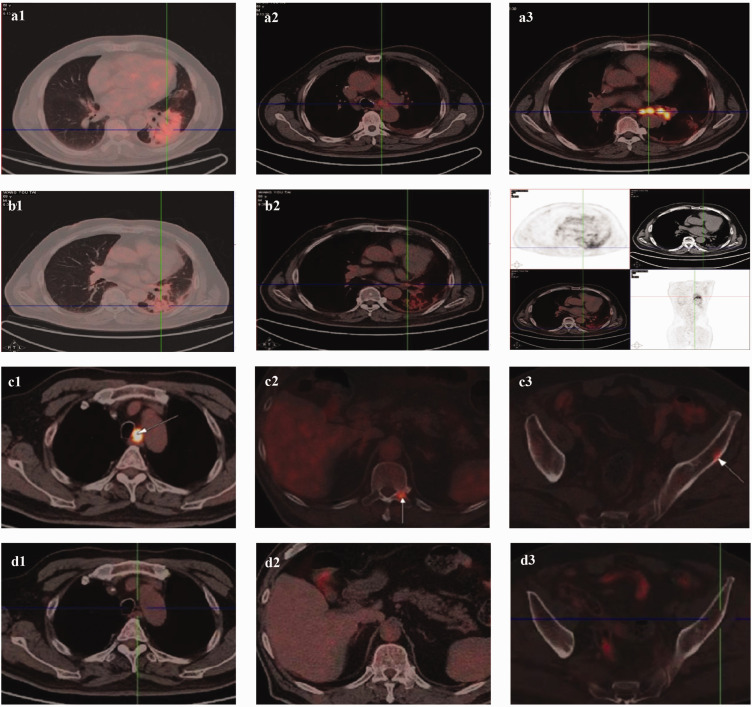
(a) Baseline chest PET/CT scan in August 2019. (a1) Lesion in the lower lobe. (a2) Lesion in the mediastinum. (a3) Lesion in the left hilum and left main bronchi. (b) PET/CT scan showing a new shadow in the lower lobe of the left lung in May 2020. (b1) Lesion in the lung window. (b2) Lesion in the mediastinal window. (b3) Lesion in all windows. (c) PET/CT in March 2021 showing (c1) enlarged lymph nodes in the left posterior trachea of the mediastinum, as well as new lesions in the (c2) left pedicle of the 11th and 12th thoracic vertebrae and (c3) left iliac wing. (d) PET/CT in April 2021 showing the lesions after two cycles of paclitaxel (albumin-bound) and carboplatin combined with the PD-L1 inhibitor durvalumab. Lesions in the (d1) mediastinum, (d2) left pedicle of the 11th and 12th thoracic vertebrae, and (d3) left iliac wing are compared with those from March 2021 and (e) PET/CT in October 2021 showed new lesions in the (e1) mediastinum, (e2) hilum, and (e3) lower lobe of the left lung.

To evaluate the tumor, positron emission tomography/CT (PET/CT) was performed, revealing consolidation in the lower lobe of the left lung with a maximum standardized uptake value of 18.6. The scan also revealed multinodular pleural involvement and increased fluorodeoxyglucose (FDG) metabolism in the lymph nodes of the mediastinum, left hilum, and left main bronchi (Figure 3(a1)–(a3)). Based on these findings, the patient was diagnosed with LS-SCLC of the left lung, with lymph node metastases to the left main bronchi, left hilum, and mediastinum, along with minimal left pleural effusion. The patient was considered to have a poor prognosis given this SCLC pathology. The lesion in the lower lobe of the left lung was considered to represent bacterial infection.

**Figure 4.**
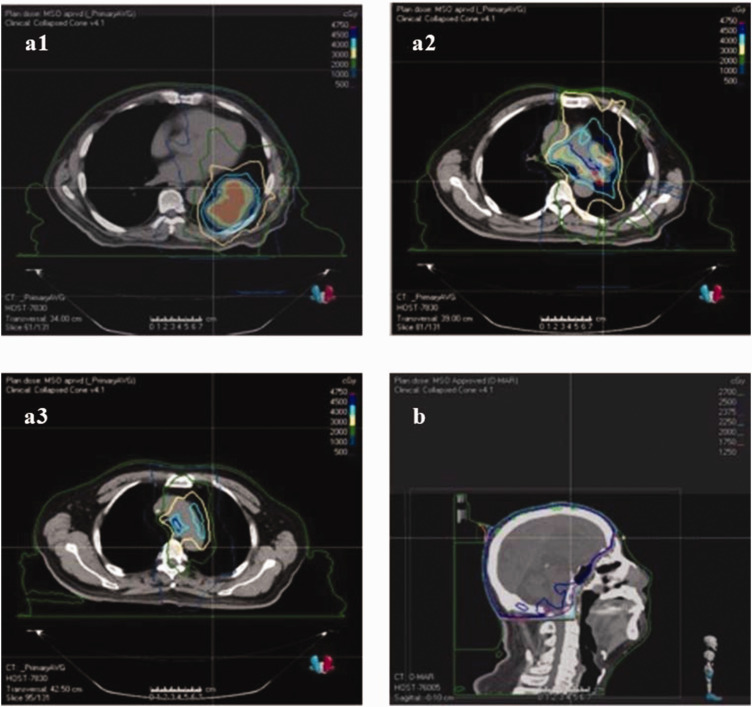
(a) Radiation field images from VMAT targeting the lesions in the (a1) lower lobe of the left lung, (a2) lymph node in the left hilum, and (a3) lymph node in the mediastinum and (b) radiation field image for PCI.

After providing informed consent to treatment, the patient received first-line therapy for LS-SCLC consisting of six cycles of chemotherapy with etoposide (100 mg intravenously (i.v.) once a day for 5 days) and carboplatin (600 mg i.v. on day 1), administered every 21 days (EC therapy). Following three cycles of chemotherapy, the patient underwent radiotherapy targeting the lesions in the lower lobe of the left lung, hilar lymph nodes, and mediastinum using volumetric-modulated arc therapy (VMAT) (6 MV X-ray, total dose: 4500 cGy in 15 fractions of 300 cGy each) (Figure 4(a1)–(a3)).

The treatment response was evaluated as partial response (PR) after two cycles of chemotherapy (Figure 2(b)) and complete response (CR) after four cycles (Figure 2(c)), with a progression-free survival (PFS) of 6 months. The NSE level normalized after two cycles of chemotherapy. Grade II myelosuppression of white blood cells occurred after two cycles of EC chemotherapy and was managed with pegylated recombinant human granulocyte colony-stimulating factor (PEG-rhG-CSF) injections (3 mg i.v. administered once a day for 1 day after every third cycle of chemotherapy). Craniocerebral magnetic resonance imaging revealed no metastatic lesions in the brain. To reduce the risk of central nervous system recurrence, prophylactic cranial irradiation (PCI) was administered (6 MV X-ray, total dose: 2500 cGy in 10 fractions of 250 cGy each) (Figure 4(b)).

Two months after completing radiotherapy, follow-up chest CT and PET/CT revealed CR of the lesion. Four months later, however, chest CT and PET/CT detected a new shadow in the lower lobe of the left lung with a maximum standardized uptake value of 6.8 (Figures 2(d) and 3(b1)–(b3)). This finding was diagnosed radiation pneumonitis. The condition was classified as grade 0 because the patient exhibited no symptoms or clinical signs. Consequently, no therapy was administered, and the condition was continuously monitored without progression. As maintenance therapy, anlotinib was initiated at a dose of 8 mg orally once a day for 14 days in a 21-day cycle. The lesions remained in complete remission, with a PFS of 20 months. No adverse reactions were observed during treatment.

At that time, chest CT revealed a new lesion in the mediastinal lymph nodes (Figure 2(e)). PET/CT showed enlarged lymph nodes in the left posterior tracheal region of the mediastinum, along with new lesions in the left pedicle of the 11th and 12th thoracic vertebrae and the left iliac wing, all exhibiting high FDG metabolism. These lesions were identified as new metastases (Figure 3(c1)–(c3)). Conversely, the shadow in the left lung had faded, with a reduction in FDG uptake. Laboratory tests showed an elevated pro-GRP level of 93.13 pg/mL, exceeding the reference range, whereas the NSE level remained within normal limits.

The patient subsequently underwent six cycles of chemotherapy combined with immunotherapy, including 400 mg albumin-bound paclitaxel (ab-paclitaxel) i.v. on day 1, 600 mg carboplatin i.v. on day 1, and 1500 mg durvalumab (a programmed death-ligand 1 (PD-L1) inhibitor) i.v. on day 1, administered every 21 days for LS-SCLC. No side effects were observed. Following two cycles, all metastatic lesions in the mediastinum, thoracic vertebrae, and iliac wing had disappeared, with the curative effect evaluation indicating CR (Figures 2(f) and 3(d1)–(d3)). CR was maintained after six cycles, and the pro-GRP level normalized after two cycles. As maintenance therapy, four additional cycles of 1500 mg durvalumab i.v. on day 1 every 21 days were administered. The patient developed no adverse reactions, and CR was sustained with a PFS of 7 months.

Next, chest CT revealed new lesions in the mediastinum, hilum, and lower lobe of the left lung (Figure 2(g) and (h)). PET/CT also showed that these lesions had high FDG metabolism, confirming them as new metastases (Figure 3(e1)–(e3)). The pro-GRP level rose to 92.19 pg/mL. The patient underwent six cycles of chemotherapy, comprising 100 mg etoposide i.v. once a day for 5 days, 30 mg cisplatin i.v. once a day for 4 days, and 1500 mg durvalumab i.v. once on day 1, administered every 21 days. Grade III myelosuppression of white blood cells was successfully managed with PEG-rhG-CSF (3 mg i.v. once for 1 day), resulting in recovery. After three cycles, all lesions achieved CR (Figure 2(i) and (j)), with a PFS of 4 months to date. The pro-GRP level normalized after the first cycle.

The reporting of this study conforms to the CARE guidelines.^5^ The patient provided written informed consent for publication of this report.

## Discussion

Lung cancer remains the leading cause of cancer-related death worldwide.^6^ Among its histological subtypes, SCLC is particularly aggressive, with a median survival duration of only 16 to 22 months and a 5-year survival rate of <20%.^7,8^ Unlike non-small cell lung cancer, SCLC is characterized by a lack of actionable mutations, leaving no effective targeted therapies available and significantly slowing therapeutic progress. Therefore, prolonging OS, enhancing quality of life, and improving the prognosis are critical goals in the comprehensive management of SCLC.

Regarding the treatment of LS-SCLC, stage I–IIA (T1–T2N0M0) accounts for <5% of cases, and only these patients are candidates for surgery. For all other patients, chemotherapy combined with VMAT is the standard treatment, with EP as the first-line chemotherapy regimen. After achieving CR or PR, PCI is recommended to prevent brain metastases. Although SCLC is sensitive to chemotherapy, most patients experience relapse within 1 year, sometimes even within 6 months.

With advancements in immunotherapy, some studies offer hope for improving the poor prognosis of SCLC. Notably, the ADRIATIC study showed promising results for durvalumab after chemotherapy with concurrent chemoradiotherapy (CCRT) in LS-SCLC. In the study, durvalumab following CCRT in LS-SCLC. The study demonstrated that durvalumab with CCRT achieved significant clinical efficacy with a tolerable safety profile.^9^ Specifically, durvalumab following CCRT resulted in a median OS of 55.9 months, extending the 2-year OS rates and reducing the risk of death by 27%. The 3-year OS rate reached 56.5%, with a 2-year PFS rate of 46.2%, compared with 47.6% and 34.2%, respectively, in the CCRT-only group. Additionally, durvalumab improved OS and PFS regardless of whether PCI was administered.^10^ Importantly, the safety profiles of both groups were similar.^11^ These findings suggest that durvalumab as consolidation therapy after CCRT represents a potential breakthrough in LS-SCLC therapy.

For second-line therapy, topotecan remains the only approved drug, although its significant toxicity, poor response rate of 24%, and median OS of only 6 months limit its utility.^4^ Ab-paclitaxel-based chemotherapy may be a better choice for recurrent SCLC, prolonging OS to approximately 8 months while reducing adverse reactions.^12,13^ Immunotherapy is becoming increasingly recognized as an essential component of lung cancer therapy. Recent studies have highlighted the synergistic activity of immune checkpoint inhibitors (ICIs) with standard platinum-based chemotherapy for extensive-stage SCLC (ES-SCLC). The CASPIAN and IMpower133 studies demonstrated the effectiveness of combining a PD-L1 inhibitor with chemotherapy in first-line treatment for ES-SCLC, achieving a median OS of approximately 13 months.^14,15^ However, there is still no evidence supporting ICI use as second-line therapy for recurrent LS-SCLC. While ICIs show promise in first-line treatment, their efficacy in second- and higher-line therapy is generally disappointing.

Currently, third-line monotherapy with nivolumab or pembrolizumab (both anti-PD-1 agents) is approved by the Food and Drug Administration for metastatic SCLC, independent of PD-L1 expression. However, this is based on limited data, and European Medicines Agency approval is lacking.^16,17^ Efforts to incorporate ICIs in combination with chemotherapy as first-line treatment for LS-SCLC are ongoing, but no results have been reported for recurrent LS-SCLC.^4^

Although SCLC lacks actionable mutation targets, the antiangiogenic drug anlotinib has been approved for third- and higher-line therapy. Anlotinib is a newly developed oral small-molecule RTK inhibitor that targets VEGFR1, VEGFR2/KDR, VEGFR3, c-Kit, PDGFR-α, and fibroblast growth factor receptors (FGFR1, FGFR2, and FGFR3). Furthermore, it inhibits both tumor angiogenesis and tumor cell proliferation.^18^ In 2019, anlotinib was approved by the NMPA for third-line therapy in SCLC. In a single-arm phase II trial, anlotinib was evaluated as part of first-line treatment for ES-SCLC, where it was combined with EP during initial therapy or used as maintenance therapy following chemotherapy. The results demonstrated a median PFS of 9.41 months and a median OS of 13.87 months, with an objective response rate of 88.89% and a disease control rate of 97.22%. These findings highlight the flexibility of combining anlotinib with various platinum-based regimens for ES-SCLC.^19^

Beyond novel treatment strategies for SCLC, methods to evaluate therapeutic efficacy and clinical outcomes are critical. Dynamic variations in serum ctDNA have been successfully used in multiple studies to evaluate therapeutic efficacy and predict OS in patients with SCLC.^20^ We will continue to monitor serum ctDNA levels in future clinical work to evaluate subsequent treatment efficacy.

In the present study, the patient initially received six cycles of first-line chemotherapy combined with VMAT, achieving CR. Anlotinib was then used as maintenance therapy, providing a PFS of 20 months, which substantially exceeded the PFS reported for standard first-line therapy. Upon recurrence, we administered six cycles of ab-paclitaxel and carboplatin combined with durvalumab, demonstrating high therapeutic efficacy and maintaining CR. The patient subsequently benefited from EP combined with durvalumab as third-line therapy. Under this comprehensive treatment strategy, this patient with LS-SCLC experienced an improved quality of life, remaining symptom-free for more than 32 months to date.

## Data Availability

The data supporting this case report are available from the corresponding author on reasonable request, in compliance with the Ethics Committee’s guidelines to ensure patient confidentiality.

## References

[1] Van Meerbeeck JP Fennell DA De Ruysscher DKM. Small-cell lung cancer. Lancet 2011; 378: 1741–1755. 21565397 10.1016/S0140-6736(11)60165-7

[2] Rami-Porta R. Staging Handbook in Thoracic Oncology. 2nd ed; IASLC: Denver, CO, USA, 2016.

[3] De Castro Carpeño J Dols MC Gomez MD , et al. Survival outcomes in stage IV small-cell lung cancer (IV-SCLC): analysis from SEER Database. Ann Oncol 2019 30: 16–32.30346470

[4] Noronha V Sekhar A Patil VM , et al. Systemic therapy for limited stage small cell lung carcinoma. J Thorac Dis 2020; 12: 6275–6290. 33209466 10.21037/jtd-2019-sclc-11 PMC7656383

[5] Gagnier JJ Kienle G Altman DG , CARE Group et al. The CARE guidelines: consensus-based clinical case reporting guideline development. Headache 2013; 53: 1541–1547. 24266334 10.1111/head.12246

[6] Cheng TY Cramb SM Baade PD , et al. The international epidemiology of lung cancer: latest trends, disparities, and tumor characteristics. J Thorac Oncol 2016; 11: 1653–1671. 27364315 10.1016/j.jtho.2016.05.021 PMC5512876

[7] Manoharan P Salem A Mistry H , et al. ^18^F-Fludeoxyglucose PET/CT in SCLC: analysis of the CONVERT randomized controlled trial. J Thorac Oncol 2019; 14: 1296–1305. 31002954 10.1016/j.jtho.2019.03.023 PMC6616906

[8] Von Pawel J Schiller JH Shepherd FA , et al. Topotecan versus cyclophosphamide, doxorubicin, and vincristine for the treatment of recurrent small-cell lung cancer. J Clin Oncol 1999; 17: 658–667. 10080612 10.1200/JCO.1999.17.2.658

[9] Cheng Y Spigel DR Cho BC , et al. Durvalumab after chemoradiotherapy in limited-stage small-cell lung cancer. N Engl J Med 2024; 391: 1313–1327. DOI: 10.1056/NEJMoa2404873. 39268857 10.1056/NEJMoa2404873

[10] Durvalumab (D) as consolidation therapy in limited-stage SCLC (LS-SCLC): outcomes by prior concurrent chemoradiotherapy (cCRT) regimen and prophylactic cranial irradiation (PCI) *Use in the ADRIATIC Trial. 2024ESMO LBA81.* 10.1016/j.jtho.2026.103703 41921748

[11] Novello S Cheng Y Spigel D , et al. Patient-reported outcomes (PROs) with consolidation durvalumab versus placebo following cCRT in limited-stage SCLC: ADRIATIC.2024 WCLC.MA17.04. 10.1016/j.jtho.2026.103564 41621752

[12] Ramalingam SS Foster J Gooding W , et al. Phase 2 study of irinotecan and paclitaxel in patients with recurrent or refractory small cell lung cancer. Cancer 2010; 116: 1344–1349. 20082454 10.1002/cncr.24753

[13] MariaAB CS Antonio R. Treatment of advanced non-small cell lung cancer. J Thorac Dis 2011; 3: 122–133. 22263075 10.3978/j.issn.2072-1439.2010.12.08 PMC3256511

[14] Goldman JW Dvorkin M Chen Y ; CASPIAN investigators et al. Durvalumab with or without tremelimumab, plus platinum-etoposide versus platinum-etoposide alone in first-line treatment of extensive-stage small-cell lung cancer (CASPIAN): updated results from a randomised, controlled, open-label, phase 3 trial. Lancet Oncol 2021; 22: 51–65. 33285097 10.1016/S1470-2045(20)30539-8

[15] Co D Paolo B Stefania C , et al. Novel cytotoxic chemotherapies in small cell lung carcinoma. Cancers (Basel) 2021; 13: 1152–1168. 33800236 10.3390/cancers13051152 PMC7962524

[16] FDA grants nivolumab accelerated approval for third-line treatment of metastatic small cell lung cancer. FDA. 2019 Sep 2 [cited 2020 Mar 8]. Available online: http://www.fda.gov/drugs/resources-information-approved-drugs/fda-grants-nivolumab-accelerated-approval-third-line-treatment-metastatic-small-cell-lung-cancer.

[17] FDA approves pembrolizumab for metastatic small cell lung cancer. FDA [Internet]. 2019 Dec 20 [cited 2020 Mar 8]. Available online: http://www.fda.gov/drugs/resources-information-approved-drugs/fda-approves-pembrolizumab-metastatic-small-cell-lung-cancer.

[18] Micke P Faldum A Metz T , et al. Staging small cell lung cancer: Veterans Administration Lung Study Group versus International Association for the Study of Lung Cancer- what limits limited disease. Lung Cancer 2002; 37: 271–276. 12234695 10.1016/s0169-5002(02)00072-7

[19] Pengbo D Huaping Y Cen C. Anlotinib plus platinum-etoposide in 1st-line treatment of extensive-stage small-cell lung cancer: a single-arm phase II trial. Worldwide Virtual Event 2021; 13: 28–31.

[20] Pellini B Chaudhuri AA. ctDNA monitoring for small cell lung cancer: ready for prime time. Clin Cancer Res 2023; 29: 2176–2178. 37097069 10.1158/1078-0432.CCR-23-0420 PMC10330146

